# Resveratrol Mitigates Uremic Toxin-Induced Intestinal Barrier Dysfunction in Chronic Kidney Disease by Promoting Mitophagy and Inhibiting Apoptosis Pathways

**DOI:** 10.7150/ijms.100963

**Published:** 2024-09-16

**Authors:** Cai-Mei Zheng, Yi-Chou Hou, Kuo-Wang Tsai, Wan-Chung Hu, Hsiu-Chien Yang, Min-Tser Liao, Kuo-Cheng Lu

**Affiliations:** 1 Division of Nephrology, Department of Internal Medicine, Shuang Ho Hospital, Taipei Medical University, New Taipei City, Taiwan.; 2 Division of Nephrology, Department of Internal Medicine, School of Medicine, College of Medicine, Taipei Medical University, Taipei, Taiwan.; 3 TMU Research Centre of Urology and Kidney, Taipei Medical University, Taipei, Taiwan.; 4 Division of Nephrology, Department of Internal Medicine, Cardinal Tien Hospital, School of Medicine, Fu Jen Catholic University, New Taipei City, Taiwan.; 5 Department of Medical Research, Taipei Tzu Chi Hospital, Buddhist Tzu Chi Medical Foundation, New Taipei City, Taiwan.; 6 Department of Clinical Pathology and Medical Research, Taipei Tzu Chi Hospital, Buddhist Tzu Chi Medical Foundation, New Taipei City 231, Taiwan.; 7 Division of Nephrology, Department of Internal Medicine, Zuoying Branch of Kaohsiung Armed Forces General Hospital, Kaohsiung 813, Taiwan; 8 Division of Nephrology, Department of Medicine, Tri-Service General Hospital, National Defense Medical Center, Taipei 114, Taiwan.; 9 Department of Pediatrics, Taoyuan Armed Forces General Hospital, Taoyuan 325, Taiwan.; 10 Department of Pediatrics, Tri-Service General Hospital, National Defense Medical Center, Taipei 114, Taiwan.; 11 Division of Nephrology, Department of Medicine, Taipei Tzu Chi Hospital, Buddhist Tzu Chi Medical Foundation, New Taipei City, Taiwan.; 12 Division of Nephrology, Department of Medicine, Fu Jen Catholic University Hospital, School of Medicine, Fu Jen Catholic University, New Taipei City, Taiwan.

**Keywords:** indoxyl sulfate, resveratrol, intestinal barrier, tight junction proteins, mitophagy, apoptosis, autophagy

## Abstract

**Background:** Chronic Kidney Disease (CKD) is a systemic progressive disorder related to uremic toxins. Uremic toxins disturb intestinal epithelial destruction and barrier dysfunction leading to gut-renal axis disorders in CKD. We examine the protective role of Resveratrol (RSV) against uremic toxin indoxyl sulphate (IS) related intestinal barrier disturbances among CKD. Methods: 5/6 nephrectomized mice and isolated primary mouse intestinal epithelial cells (IEC-6) are used to assess the influence of IS on intestinal epithelial tight junction barriers. Serum biochemistry parameters, hematoxylin & eosin (H&E) and immunohistochemistry staining (IHC), Western blot analysis, q-PCR, and si-RNA targeted against AhR were used in this study. Results: IS decreases the expression of tight junction proteins (TJPs) ZO-1 and claudins, increases the apoptosis and impairs mitophagy within IECs. Treatment with RSV not only reduces the loss of TJPs but also modulates mitophagy markers LC3 and P62, and concurrently decreases the levels of apoptosis-related proteins. Significantly, RSV ameliorates intestinal barrier dysfunction in CKD by modulating mitophagy via the IRF1-DRP1 axis, restoring autophagy, and inhibiting apoptosis through the activation of the PI3K/Akt-Ho-1 anti-oxidant pathway, and mTOR regulated pathways. Conclusion: This study establishes RSV as a potential therapeutic agent that can ameliorate gut-renal axis disturbances in CKD. These findings provide valuable insights into mechanisms underlying RSV RSV-mediated gut-renal axis, highlighting its effectiveness as a potential treatment option for CKD-associated intestinal barrier dysfunction.

## Introduction

Worldwide, chronic kidney disease (CKD) is a major health concern, affecting more than 800 million individuals, or over 10% of the global population 1. The gut-kidney axis is increasingly recognized for its critical role in CKD development and progression, due to the complex interplay between intestinal and renal functions. CKD patients frequently suffer from gastrointestinal issues including anorexia, diarrhea, constipation, and mucosal bleeding 2. Conversely, intestinal problems can exacerbate kidney damage through systemic inflammation, altered gut microbiota, and the production of uremic toxins such as indoxyl sulfate (IS) and p-cresyl sulfate (PCS) 3. The interaction within the gut-kidney axis in CKD is primarily associated with disruptions in metabolic and immune pathways 4. Uremic toxins play an important role in the interplay between these metabolic and immune pathways among CKD patients 5. Additionally, structural impairments such as increased intestinal permeability and weakened intestinal barriers were observed in both humans and animals suffering from advanced CKD 6, 7. Vaziri et al. revealed that uremia negatively impacts intestinal tight junctions by a significant reduction in junctional proteins including occludin and claudin-1 levels 8. The exploration of the mechanisms underlying the gut-renal axis is critical for novel therapeutic strategies.

Recent studies proved that major uremic toxin IS implicated in various deleterious processes within the body through activation of Aryl Hydrocarbon Receptor (AhR) related immune and inflammatory responses 9, 10. The aryl hydrocarbon receptor (AhR)- nuclear factor erythroid 2-related factor 2 (Nrf2) pathway was proved to relate with intestinal epithelial tight junction proteins 11. Huang et al demonstrated that IS plays a critical role in CKD-related intestinal barrier injuries 12. Targeting the uremic toxins related to intestinal barrier disruption and mitophagy pathways within intestinal epithelial cells might represent new therapeutic strategies for gut-kidney axis disturbances in CKD.

Resveratrol (RSV), a 3,5,4'-trihydroxy-trans-stilbene, an antagonist of AhR, exerts its' protective effects in various disease models through anti-inflammatory and anti-oxidant properties 13. RSV can reduce serum uremic toxin levels by inhibiting hepatic sulfotransferase and gut microbial trimethylamine-N-oxide (TMAO) production 14 which alters the favorable microbial profile within the intestine 15, 16, which are critical for intestinal barrier dysfunction. While RSV shows potential therapeutic benefits, its effects on uremia-induced intestinal barrier dysfunction in CKD are not fully explored 16. Animal studies suggest RSV protects intestinal cells from oxidative stress via the PI3K/Akt-Nrf2 pathway, maintaining barrier integrity 17. This study aims to investigate RSV's protective effects against IS-induced intestinal damage and explore the underlying mechanisms, particularly focusing on mitophagy and apoptosis signaling pathways, to provide insights into potential CKD management strategies.

## Materials and Methods

### Reagents and Chemicals

Culture media components like DMEM, fetal bovine serum, and antibiotic-antimycotic (100×), were acquired from Gibco (Las Vegas, NV, USA). The indoxyl sulfate (IS) and resveratrol (RES) were obtained from Sigma (St. Louis, MO, USA). Primary antibodies as follows: Anti-ZO-1(Cell Signaling Technology, Danvers, MA, USA), anti-Occludin (Cell Signaling Technology, Danvers, MA, USA), anti-Occludin-1 (Cell Signaling Technology, Danvers, MA, USA), anti-Occludin-2 (Cell Signaling Technology, Danvers, MA, USA), anti-IRF-1 (GeneTex, Hsinchu City, Taiwan), anti-DRP-1 (Cell Signaling Technology, Danvers, MA, USA), anti-Ho-1(Cell Signaling Technology, Danvers, MA, USA), anti-Bax (Cell Signaling Technology, Danvers, MA, USA), anti-Bcl-2 (Cell Signaling Technology, Danvers, MA, USA), anti-cytochrome C (Abcam, Cambridge, UK), anti-caspase 3 (Cell Signaling Technology, Danvers, MA, USA), anti-PARP-1 (Cell Signaling Technology, Danvers, MA, USA), anti-LC3 A/B (Cell Signaling Technology, Danvers, MA, USA), anti-P62 (Cell Signaling Technology, Danvers, MA, USA), anti-mTOR (Cell Signaling Technology, Danvers, MA, USA) and anti-PI3K/AKT (Cell Signaling Technology, Danvers, MA, USA), and anti-β Actin (Proteintech, Rosemont, IL, USA). Horse Radish peroxidase (HRP)-conjugated anti-rabbit immunoglobulin G and anti-mouse immunoglobulin G were obtained from the Proteintech Group (Chicago, IL, USA). Real-time PCR, and qPCR reagents were obtained: RNA Extractor kit (Tools, Taiwan), DNase I Amplification Kit (Invitrogen, USA), iScript cDNA Synthesis Kit (Bio-Rad, Hercules, CA, USA), and Simply Green qPCR Master Mix (GeneDireX Inc., Taichung City, Taiwan).

### *In-Vivo* Study

**Figure 1:**
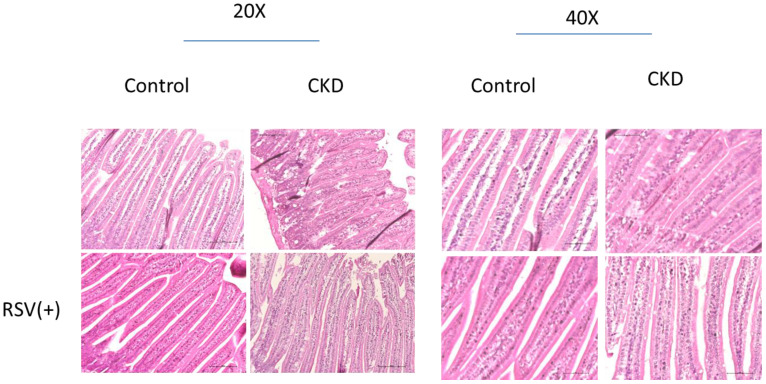
Histological assessment of intestinal morphology in a mouse model of chronic kidney disease (CKD) with and without resveratrol (RSV) treatment. The left panel shows 20x magnification, and the right panel shows 40x magnification. In the CKD models, disorganization and irregularity of the epithelial structure are evident as compared to controls. With RSV treatment, a reestablishment of the epithelial architecture towards a more organized and uniform structure is observed, indicating a protective or restorative effect of RSV on intestinal morphology in CKD.

Institutional Animal Care and Use Committee (110-IACUC-001, 2021) approved the animal studies and all were conducted following National Institutes of Health Guidelines. BALB/c male mice (6 weeks old with a body weight of more than 20 g) with 5/6 nephrectomy (Nx) or sham surgery were obtained from BioLASCO Taiwan Co., Ltd (Taipei, Taiwan) and divided into four groups (control (n = 6), sham + resveratrol (RSV) injection (n = 6), 5/6 Nx + N/S injection (n = 6), and 5/6 nephrectomy + resveratrol (RSV) injection (n = 6)) (110-IACUC-001, 2021). 5/6 nephrectomy surgery was done when the mice were 4 weeks old; firstly, 2/3 of the right-side kidney was removed; one week later, the whole left side of the kidney was removed. Sham mice only received a back incision with open & close surgery. All mice were kept in pathogen-free animal facilities under the same conditions of 22 °C and 12 h light/dark cycle. At 10 weeks of age, 5/6 nephrectomy mice had significantly higher serum creatinine and BUN levels than the control group (Figure 1A, B). The mice received an intraperitoneal (IP) injection with N/S (110 λ/mouse) or an RSV (30 mg/kg/day, about 110 λ/mouse) for 4 weeks. Then, the mice were sacrificed, and their bones were analyzed.

### Serum biochemistry analysis

Blood specimens were drawn from the mice's tail vein and deposited into heparinized microtubes. Subsequently, serum was separated via centrifugation at 1000 g for 20 minutes at 4 °C. The serum was then subjected to analysis over two hours. Serum creatinine (SCr) and blood urea nitrogen (BUN) levels were quantified using the biochemical analysis facilities at the Taiwan Mouse Clinical (National Phenotyping Center, Taipei, Taiwan).

### H&E and IHC staining

The specimen was preserved in 4% paraformaldehyde, subjected to graded ethanol dehydration, and embedded in paraffin wax. Subsequently, 5 µm sections were cut longitudinally. For Hematoxylin and Eosin (H&E) staining, sections underwent deparaffinization and rehydration, followed by staining in hematoxylin for 5 minutes and eosin for 3 minutes (ScyTek Laboratories, Logan, UT, USA). For immunohistochemical (IHC) analysis, sections were antigen-retrieved in a 60 °C water bath for 24 hours, then incubated with primary antibodies against Zo-1 (1:500), Claudin-2 (1:1000), and Cytochrome C (1:500) overnight at 4 °C. Subsequent detection employed Rabbit Probe HRP Chromogen with DAB Brown (BioTnA Biotech, Kaohsiung, Taiwan) for 30 minutes, followed by a brief hematoxylin counterstain. Microscopic evaluation was conducted using a Carl Zeiss microscope across multiple random fields per group, with quantitative analysis of stained regions using the AxioVision Measurement Program via an Axiocam MRC camera (Carl Zeiss, Germany).

### *In-Vitro* study

The IEC-6 intestinal cell line was cultured in Dulbecco's Modified Eagle Medium (DMEM) supplemented with 10% fetal bovine serum, 1% antibiotic-antimycotic solution (100x), and 0.1 Unit/ml human insulin. These cells were incubated at 37 °C within a 5% CO2 humidified environment. Vanholder et al. reported that normal IS concentration in healthy individuals is 2 μM, while CKD patients have a mean/median uremic concentration of 211 μM (53 ± 91.5 mg/L) and a maximum of 940 μM (236 mg/L) 18. Our previous research found that short-term exposure to low-dose IS (<100 μM) enhances osteoclast differentiation, whereas long-term exposure to high-dose IS (>250 μM) diminishes it 19. In this study, we exposed ICE-6 cells to four IS concentrations (0, 125, 250, and 500 μM) to assess the effects of different IS levels on ICE-6 cells. The working concentration of Resveratrol (RSV) was established from an osteoblast pilot study 20, 21. After testing various concentrations, we found that 10 μM was particularly effective, so we selected this concentration for further investigation.

### Western blot analysis

Protein extracts from IEC-6 cells were isolated using RIPA buffer supplied by Bio Basic Inc. (Toronto, Canada), which included a broad-spectrum protease inhibitor cocktail from BIONOVAS (Toronto, Canada). These protein samples were then individually placed into an SDS-PAGE gel slot, subjected to electrophoresis, and subsequently transferred onto a polyvinylidene fluoride (PVDF) membrane. This membrane was then exposed to primary antibodies at 4 °C overnight and to secondary antibodies for one hour at ambient temperature. The protein bands were visualized using an ECL substrate by GE Healthcare (Chicago, IL, USA), and the band intensity was measured using ImageJ software provided by NIH (Bethesda, MD, USA).

### qPCR and si-RNA AhR

Cellular RNA was isolated from cells with an RNA extraction kit supplied by Tools (New Taipei City, Taiwan) and its concentration was measured with a Nanodrop 2000 spectrophotometer (Thermo Fisher; Waltham, MA, USA), following established procedures. The cDNA was synthesized using an iScript cDNA Synthesis Kit. Subsequently, real-time quantitative PCR (qPCR) was performed with the LightCycler®480 Instrument II (Roche Molecular Systems, Inc.; Pleasanton, CA, USA) to assess the expression levels of osteoblast development marker genes. Details of the qPCR primers used are provided in Table 1, with all primers produced by Genomics (Taipei, Taiwan). The mRNA levels of β-actin served as an internal standard to normalize gene expression across the samples.

### Statistical analysis

Representative data are shown as mean ± standard deviation (SD) from at least three separate experiments for each condition. Statistical analyses were performed using SAS 9.0 software (SAS Institute Inc., Cary, NC, USA). A p-value of less than 0.05 was deemed statistically significant.

## Results

### RSV improves the intestinal barrier structural and functional injuries in CKD mice

5/6 nephrectomy (Nx) mice demonstrated significant disorganization of the epithelial cells, with irregular villous architecture and disrupted cellular arrangement with augmented permeability. Observable histological alterations within the intestinal tissues included reduced goblet cell populations, necrotic villi, noted edema, and instances of ulceration. Moreover, measurements of intestinal wall thickness indicated notable hypertrophy while pathologic examination revealed the accretion of mononuclear leukocytes within the lamina propria, suggesting an inflammatory response. Immunohistochemistry studies on colonic tissues revealed a decreased expression of crucial tight junction proteins, like zona occludins-1, occludin, claudin-1, and claudin-2, in both the 5/6Nx+N/S treatment group and the 5/6Nx+RSV groups when bench-marked against a control (sham) group (Figure 1 & 7). However, the decline in critical tight junction proteins—occludin, claudin-1, and claudin-2—was notably less pronounced in RSV-treated mice (p < 0.05). In brief, the administration of 10uM of Resveratrol (RSV) presented a protective effect and a notable improvement in the structural integrity of the small intestinal epithelium in CKD (Figures 1 & 8). Please note that this analysis focuses on describing qualitative morphological characteristics and does not include detailed quantitative data or comparisons with untreated controls.

### RSV increases the tight junction protein expression in IS-treated intestinal epithelial cells

**Figure 2:**
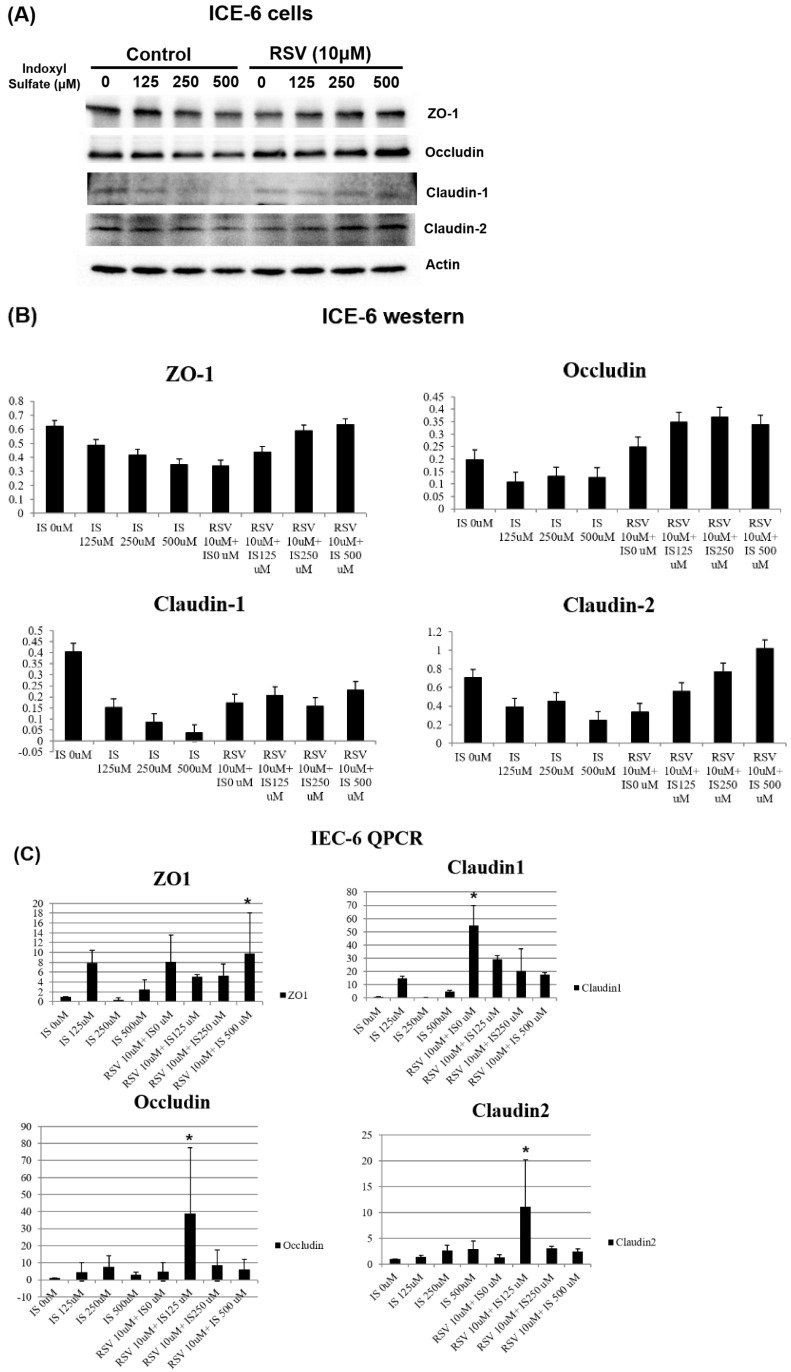
The protein expression of tight junction proteins (TJPs) in intestinal epithelial cell line IEC-6 under various conditions. The cells were treated with different concentrations of indoxyl sulfate (IS) with and without resveratrol (RSV)- 10 μM. (A) The western blot analysis revealed that higher concentrations of IS correlate with decreased expression of TJPs including ZO-1, Occludin, Claudin-1, and Claudin-2. RSV mitigates the negative impact of IS, suggesting a protective effect of RSV on TJP expression. (B) A quantitative analysis of the western blot data from (A). The bar graphs depict the relative expression levels of ZO-1, Occludin, Claudin-1, and Claudin-2. (C) qPCR analysis of mRNA expression levels for the same TJPs in IEC-6 cells. IS dose-dependently downregulates the TJP mRNA expression, which is reversed by RSV treatment.

Further, the expression of various tight junction proteins, including zona occludins 1 (ZO-1), Occludin, Claudin-1, and Claudin-2, experienced considerable suppression due to IS exposure when compared to the control. Pretreatment with 10µM of resveratrol (RSV) notably mitigated the suppressive influence of IS on ZO-1, Occludin, Claudin-1, and Claudin-2 proteins. These findings demonstrate that RSV treatment mitigates the dysfunction of the intestinal barrier by promoting the expression of TJPs both at the mRNA and protein levels in uremic conditions (Figure 2).

### RSV reverses the IS-induced impaired mitophagy in intestinal epithelial cells

**Figure 3:**
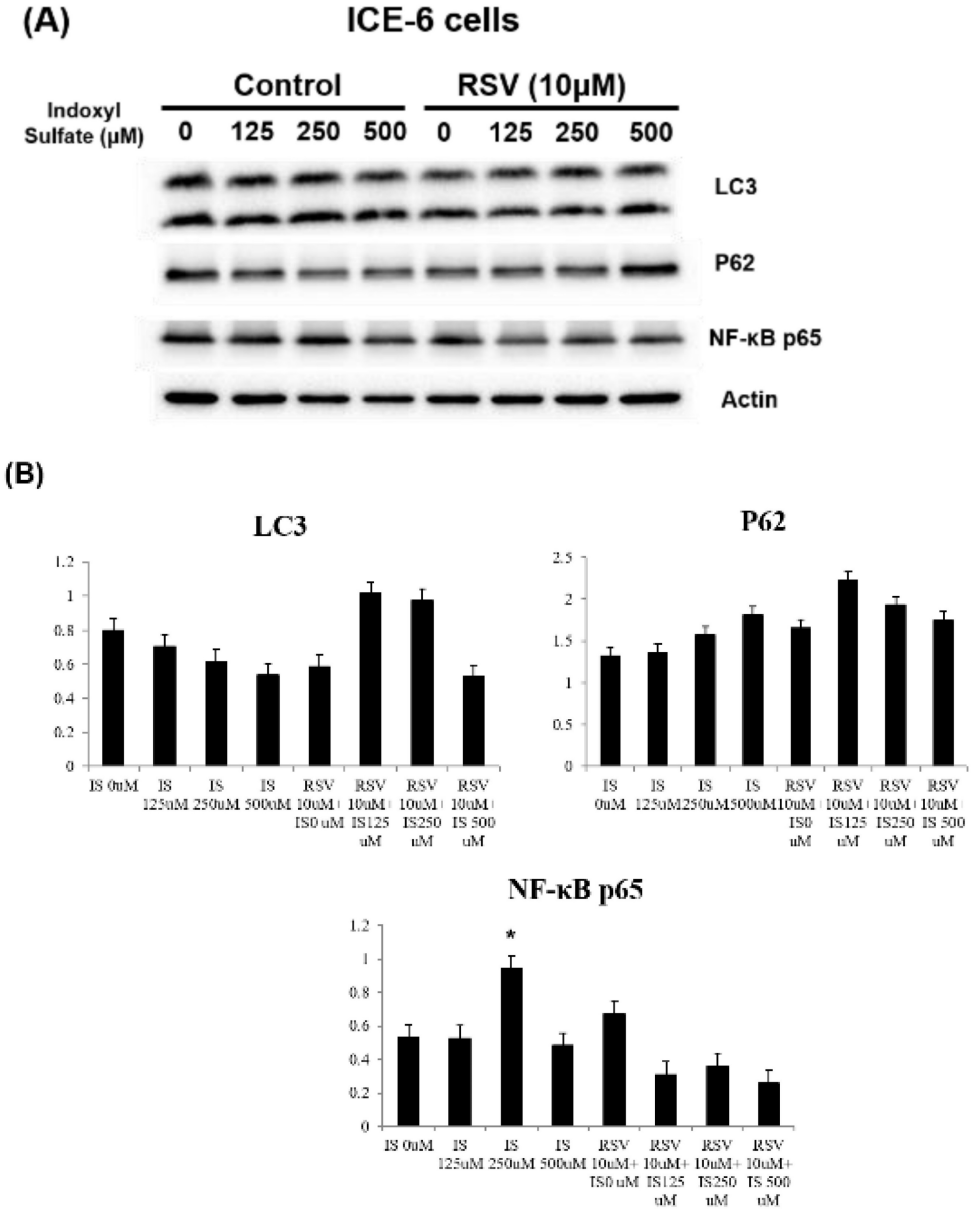
Expression of mitophagy markers in ICE-6 cells under IS with and without RSV treatment. IS treatment decreases LC3 and increases P62 levels with the favor of dysregulated mitophagy. It also increases NF-κB p65 expression and enhance inflammation. Treatment with RSV counters these effects, including an increase in LC3 levels and a decrease in NF-κB p65 expression within ICE-6 cells.

**Figure 4:**
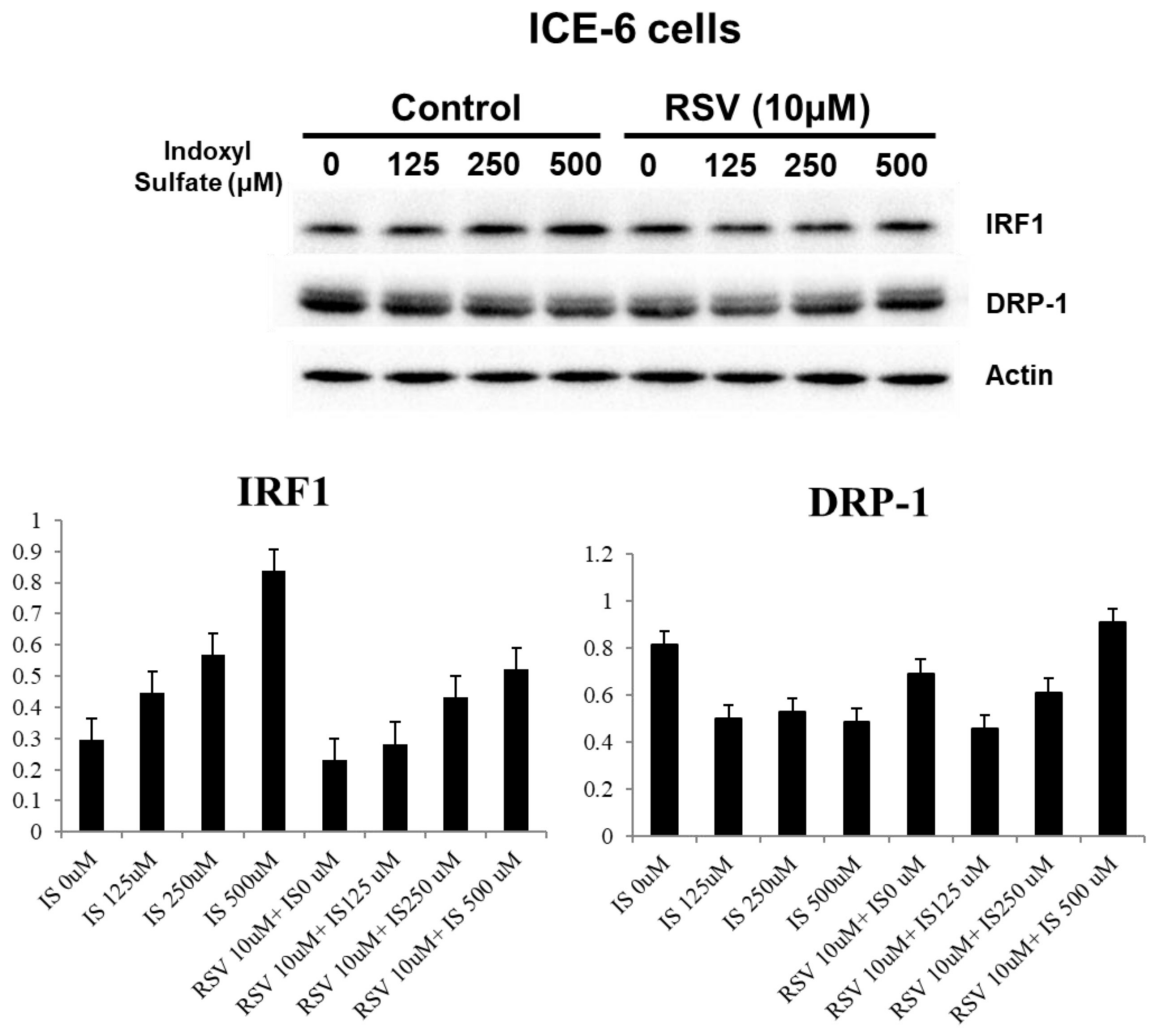
The impact of IS and RSV on expression of proteins IRF1 and DRP1 which influence the mitochondrial dynamics within intestinal epithelial cells. IS dose-dependently increases IRF1 and decreases DRP1 expression. On the other hand, 10μM RSV inhibits the IS-induced IRF1 expression and concomitantly enhances DRP1 expression.

We explored the underlying mechanisms related to indoxyl sulfate (IS) and resveratrol (RSV) within IECs. IS treatment decreases LC3 and increases P62 levels in favor of dysregulated mitophagy. It also increases NF-κB p65 expression and enhances inflammation. Treatment with RSV counters these effects, including an increase in LC3 levels and a decrease in NF-κB p65 expression within ICE-6 cells (Figure 3). This restoration of mitophagy is possibly through regulation of IRF1 and DRP1 which influence the mitochondrial dynamics within intestinal epithelial cells. IS dose-dependently increases IRF1 and decreases DRP1 expression. On the other hand, 10 μM RSV inhibits the IS-induced IRF1 expression and concomitantly enhances DRP1 expression (Figure 4).

### Resveratrol improves the IS-related apoptosis and autophagy dysregulation within intestinal epithelial cells

**Figure 5:**
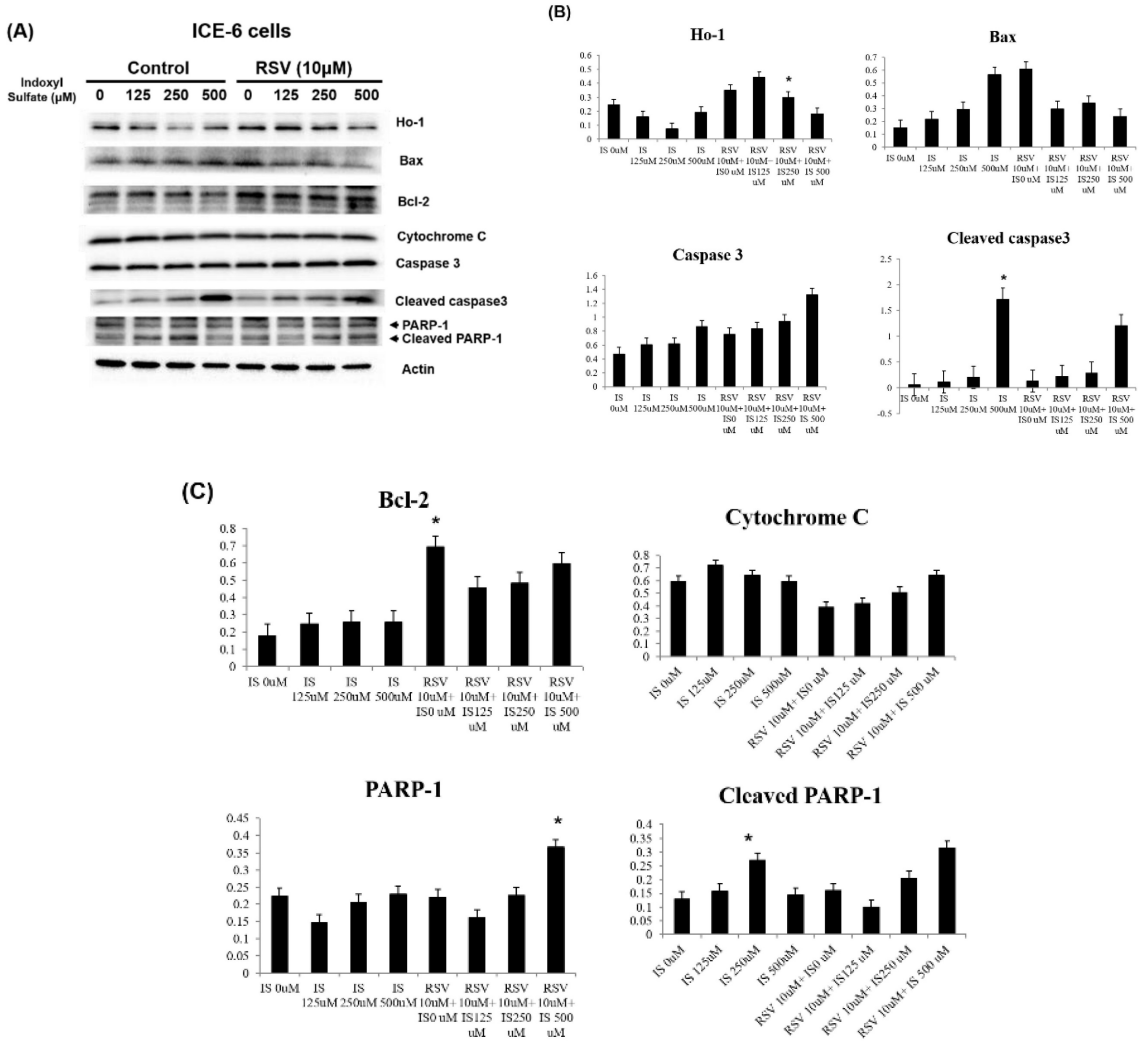
Role of IS and RSV on the expression of various apoptotic proteins within IEC-6 cells. IS increases the apoptotic proteins expression, including Bax, Cytochrome C, and cleaved PARP-1 and caspases. Conversely, RSV exerts anti-apoptotic actions through increased Bcl-2 and HO-1 expression within IS treated IEC-6 cells.

**Figure 6:**
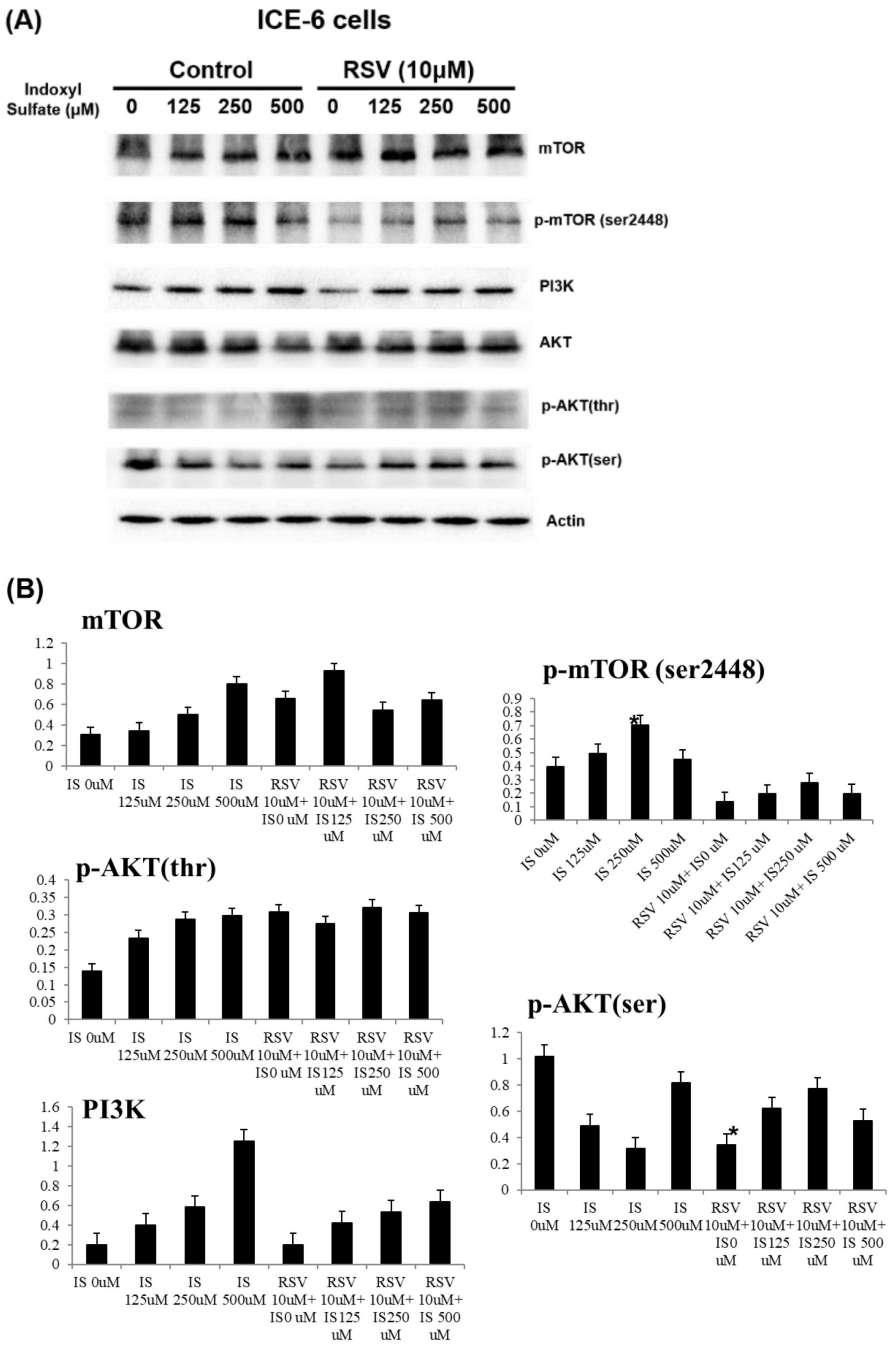
Key signaling proteins in the PI3K/AKT/mTOR pathways in ICE-6 cells treated with varying concentrations of indoxyl sulfate (IS) with and without resveratrol (RSV, 10μM). The phosphorylation of mTOR (Ser2448), a marker of mTOR activity, and AKT (on serine and threonine residues), a critical mediator of survival signaling, are significantly altered in response to IS treatment. RSV exhibits a protective effect by modulating this pathway, which may result in the restoration of autophagy and a reduction in apoptosis.

**Figure 7:**
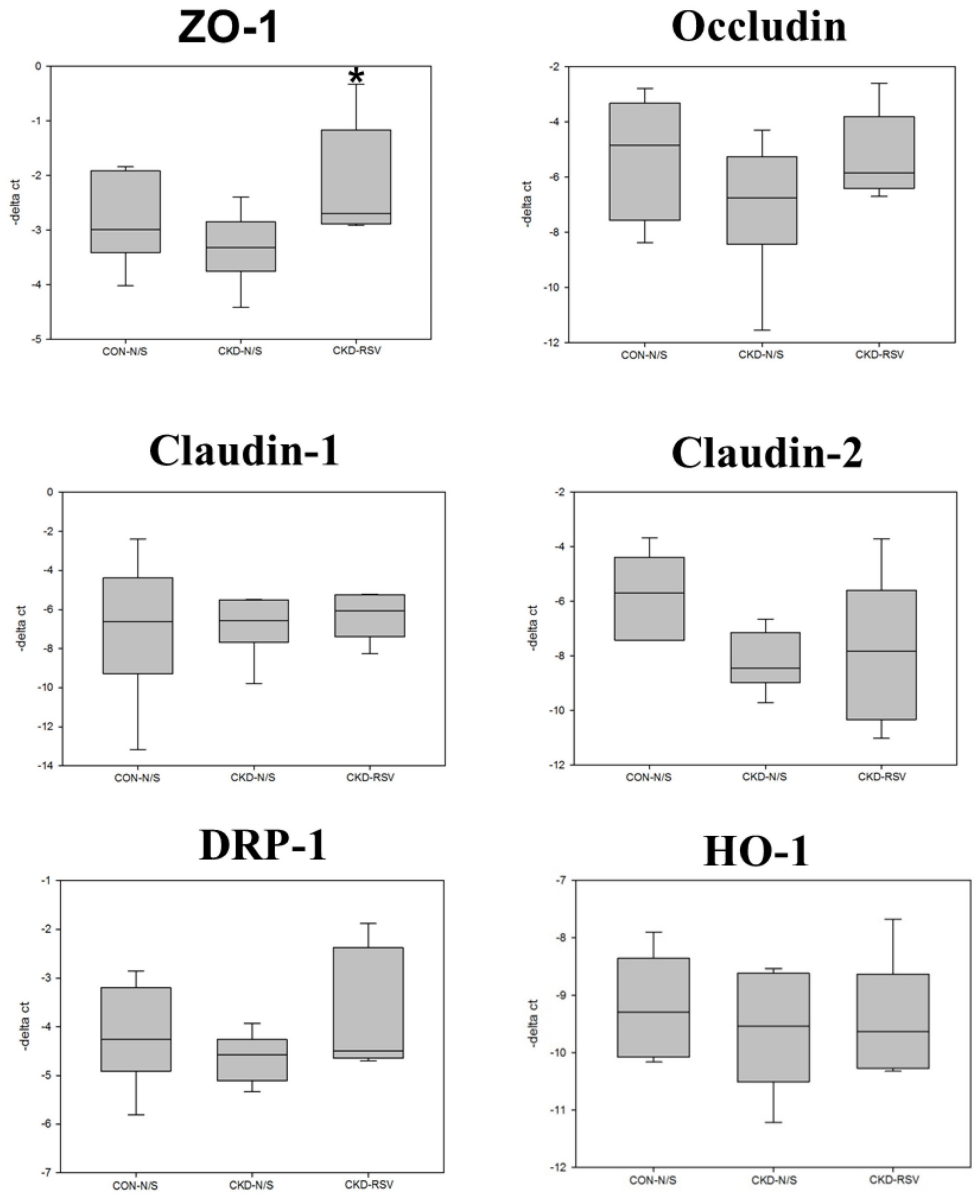
Box plots reveal the relative mRNA expression of tight junction proteins (TJPs), mitochondrial dynamics, and protective enzymes in mouse small intestinal epithelial tissue analyzed via quantitative PCR (qPCR). The TJPs examined are ZO-1, Occludin, Claudin-1, and Claudin-2. Mitochondrial dynamics protein, DRP-1 and HO-1 are also assessed. CON+NS: control non-CKD mice; CKD+NS: CKD mice without RSV treatment; CKD+RSV: CKD mice treated with RSV.

**Figure 8:**
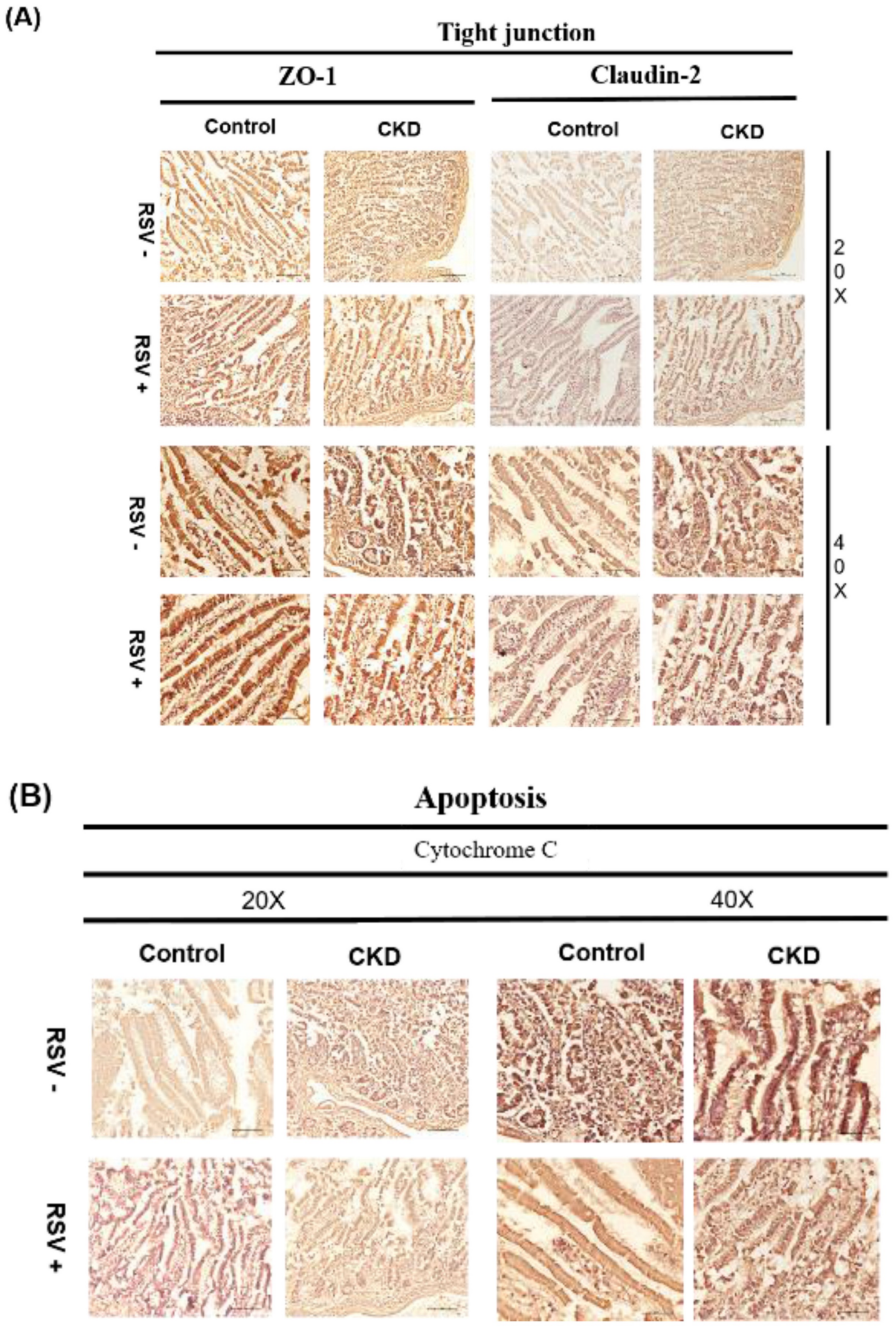
Immunohistochemical (IHC) staining for TJPs (ZO-1 and Claudin-2) and apoptotic marker cytochrome C in mouse small intestinal epithelial cells. (A) In CKD mice, a reduced expression of ZO-1 and Claudin-2 is evident, RSV treatment results in an improvement in tight junction protein expression. (B) Cytochrome C expression is increased in CKD mice, whereas RSV treatment decreases its expression.

IS enhanced the expression of pro-apoptotic proteins (Bax, cleaved caspase 3, cytochrome C, PARP-1) while reducing anti-apoptotic Bcl-2. Conversely, RSV pretreatment significantly mitigated these effects, reducing the overexpression of pro-apoptotic markers and bolstering Bcl-2 levels. This suggests that RSV can protect against cellular apoptosis induced by IS, by regulating Bcl-2/Bax/cleaved caspase 3/cytochrome c/PARP-1 and Ho-1-related pathways (Figure 5). Further, RSV modulates the PI3K/AKT/mTOR pathway in IS-treated ICE-6 cells, which may result in the restoration of autophagy and a reduction in apoptosis (Figure 6).

## Discussion

The great interest in the gut-renal axis underscores a critical paradigm shift in our understanding of chronic kidney disease (CKD) pathophysiology. Our findings elucidate the protective role of resveratrol (RSV) against indoxyl sulfate (IS)-induced disruptions within this axis, particularly focusing on intestinal barrier integrity. Uremia significantly compromises both the structural and functional coherence of the intestinal barrier, with particularly its tight junctions 6. In accordance, we found a weakened structural composition and heightened intestinal permeability in our 5/6 nephrectomy mice. From *in-vivo* and *in-vitro* studies, we proved that protein expression of TPJs was significantly suppressed by IS treatment, and RSV pretreatment retarded such suppression. In a study among high-fat diet-induced insulin-resistant mice, Chen, K. et.al., also revealed that RSV strengthens intestinal integrity by regulating tight junction protein expression 22.

Mitochondrial health is essential for maintaining epithelial barrier function, encompassing tight junctions, mucus production, antimicrobial peptides, and immune tolerance 23. Effective mitophagy reduces cellular stress and damage, maintaining tight junctions. Healthy mitochondria are essential for ATP production needed to maintain tight junctions. Damaged mitochondria increase oxidative stress and inflammation, compromising barrier function. Impaired mitophagy leads to mitochondrial dysfunction, reduced energy production, and barrier disruption. Mitophagy also helps control inflammation, further protecting tight junction integrity 24, 25. RSV is recognized for its diverse biological effects, including antioxidant, anti-inflammatory, and anti-cancer properties 26. Our previous study indicated that RSV can reverse the IS-induced decline in osteoblast development via the IS/AhR/MAPK signaling pathway in CKD mice 21. RSV prevented muscle wasting caused by a high-fat diet in older rats by restoring mitochondrial function and reducing oxidative stress through the PKA/LKB1/AMPK signaling pathway 27. In this study, we proved the effects of IS and RSV on the expression of mitophagy markers LC3 and P62 within the intestinal epithelial cells (IECs). IS treatment decreased LC3, increased P62 and NF-κB p65 expression; and improved by pre-treatment with RSV in IECs. Mitophagy plays a role in diminishing oxidative stress by removing dysfunctional mitochondria and may also safeguard against apoptosis by lessening mitochondrial outer membrane permeabilization (MOMP) and curtailing the release of pro-apoptotic mitochondrial proteins 28. Previous *in-vivo* and *in-vitro* findings revealed that IS disrupted the IRF1-DRP1 axis-related mitophagic flux 12. In our IS-treated IECs, we found an inhibition of DRP1 with enhanced IRF1 expression with decreased mitophagy proteins. We further demonstrated that RSV improved the IS-related disturbances in mitophagy and intestinal epithelium damage through IRF-DRP1 axis restoration. Our study highlights that RSV lowers IS-induced IRF1 expression, reduces DRP1 activation, and restores the balance between mitochondrial fission and fusion, thereby maintaining mitophagy and alleviating cellular stress.

Mitophagy also regulates apoptosis by maintaining mitochondrial quality. Effective mitophagy prevents the buildup of damaged mitochondria and the release of pro-apoptotic factors like cytochrome c 29. In CKD, impaired mitophagy can cause mitochondrial dysfunction, increase apoptotic signals, and promote excessive apoptosis in the intestinal epithelium 30. Compromised mitophagy leads to oxidative stress and cellular damage, activating apoptosis and worsening intestinal barrier integrity. Chronic inflammation in CKD further disrupts mitophagy and enhances apoptosis, creating a cycle that exacerbates intestinal barrier dysfunction and increases permeability, leading to more CKD-related complications 31. A surge in oxidative stress and apoptosis were important findings among IS-treated TECs alongside an increase in apoptotic protein Bax and a decrease in anti-apoptotic Bcl-2 protein levels 32, 33. Moreover, in IS-treated mice, inflammatory cells infiltrating the intestinal mucosa exhibited higher Bax expression as compared to control 32. By inhibiting apoptosis, RSV preserves the cellular environment necessary for efficient mitophagy, thus supporting mitochondrial health.

Previous studies have observed that RSV augmented mitochondrial production and improved oxidative stress through Nrf2/HO-1 pathways within the intestines 17, 34. Herein, we proved that RSV pre-treatment elevated the levels of anti-oxidative proteins including HO-1, while simultaneously reducing the levels of apoptotic proteins including Bax, cytochrome C, and cleaved caspase 3, etc., within IS-treated IECs. It has been noted that the PI3K/Akt pathway is crucial for the protective effects conferred by RSV-activated Nrf2 in response to oxidative stress 17. RSV activates the PI3K/Akt pathway to induce Ho-1, an enzyme with strong anti-apoptotic and cytoprotective properties 35. In our *in-vitro* study, RSV upregulated the levels of PI3K/p-Akt and HO-1 in IS-treated IECs, suggesting that RSV attenuated IS-induced oxidative status possibly through activating PI3K/Akt/HO-1 signaling pathways. HO-1, an enzyme crucial for defending against oxidative stress, facilitates the degradation of heme into biliverdin, carbon monoxide, and free iron. Biliverdin and its derivative bilirubin serve as potent antioxidants 36. RSV treatment led to an upregulation of HO-1 in IS-treated IECs, indicating that RSV enhances the cell's antioxidant capacity by increasing HO-1 production. We delved into the protein mTOR, an inhibitor of autophagy, and found that its expression is notably reduced in the presence of IS. Pretreatment with RSV significantly increases the p-mTOR expression, which in turn helps alleviate the IS-induced autophagy impairment within IECs. IS can impair mTOR signaling, resulting in either excessive or insufficient mitophagy 37. RSV may regulate mTOR activity, ensuring a balanced autophagic response that prevents damaged mitochondria buildup and promotes their selective degradation, thus supporting cellular health.

While animal models provide valuable insights, researchers need to acknowledge their limitations and explore more human-relevant models to enhance translation to clinical practice. Further clinical research is essential to address these limitations and advance our understanding of CKD-related intestinal barrier dysregulation 38.

In conclusion, our study revealed the therapeutic potential of RSV in ameliorating CKD-associated intestinal barrier dysfunction, highlighting its role in modulating key pathophysiological pathways within the gut-renal axis. RSV represents a promising candidate for further research and development into effective treatments for chronic kidney disease patients.

## Figures and Tables

**Table 1:** Listing of oligonucleotide primers for quantitative PCR (qPCR). "F" and "R" denote forward and reverse primers, respectively. The genes targeted by these primers include those encoding for dynamin-related protein 1 (DRP1), interferon regulatory factor 1 (IRF1), zonula occludens-1 (ZO-1), claudin-2, occludin, claudin-1, heme oxygenase-1 (HO-1), and glyceraldehyde 3-phosphate dehydrogenase (GAPDH). GAPDH is commonly used as a housekeeping gene for normalization purposes in qPCR experiments.

OligoName	Primer probe	Oligo Sequence
DRP1	F	ATGCCAGCAAGTCCACAGAA
	R	TGTTCTCGGGCAGACAGTTT
IRF1	F	TCCAAGTCCAGCCGAGACACTA
	R	ACTGCTGTGGTCATCAGGTAGG
ZO-1	F	GGGAGGGTCAAATGAAGACA
	R	GGCATTCCTGCTGGTTACAT
Claudin-2	F	GCAAACAGGCTCCGAAGATACT
	R	GAGATGATGCCCAAGTACAGAG
Occludin	F	GCCTGGACATTTTGCTCATCA
	R	CCACACAGGCAAATATGGCG
Claudin-1	F	GGCTTCTCTGGGATGGATCG
	R	CCCCAGCAGGATGCCAATTA
Ho-1	F	GGTGACAGAAGAGGCTAAGAC
	R	GCTCCTCAAACAGCTCAATG
GAPDH	F	GCATCTTCTTGTGCAGTGCC
	R	ACTGTGCCGTTGAATTTGCC
